# The association of azole antifungals with overall survival in patients with non-small cell lung cancer receiving immune checkpoint inhibitors

**DOI:** 10.1093/oncolo/oyae262

**Published:** 2024-09-25

**Authors:** Nikhil T Sebastian, William A Stokes, Madhusmita Behera, Renjian Jiang, David A Gutman, Zhonglu Huang, Abigail Burns, Vidula Sukhatme, Michael C Lowe, Suresh S Ramalingam, Vikas P Sukhatme, Drew Moghanaki

**Affiliations:** Department of Radiation Oncology, Emory University, Atlanta, GA 30322United States; Winship Cancer Institute, Emory University, Atlanta, GA 30322, United States; Department of Radiation Oncology, Emory University, Atlanta, GA 30322United States; Winship Cancer Institute, Emory University, Atlanta, GA 30322, United States; Winship Cancer Institute, Emory University, Atlanta, GA 30322, United States; Winship Cancer Institute, Emory University, Atlanta, GA 30322, United States; Winship Cancer Institute, Emory University, Atlanta, GA 30322, United States; Atlanta Veterans Affairs Health Care System, Decatur, GA 30033, United States; Winship Cancer Institute, Emory University, Atlanta, GA 30322, United States; Atlanta Veterans Affairs Health Care System, Decatur, GA 30033, United States; Morningside Center for Innovative and Affordable Medicine, Emory University, GA, Atlanta 30322, United States; GlobalCures, Inc., Newton, MA 02459, United States; Morningside Center for Innovative and Affordable Medicine, Emory University, GA, Atlanta 30322, United States; Division of Surgical Oncology, Emory University, Atlanta, United States; Winship Cancer Institute, Emory University, Atlanta, GA 30322, United States; Department of Hematology and Medical Oncology, Emory University, Atlanta, United States; Morningside Center for Innovative and Affordable Medicine, Emory University, GA, Atlanta 30322, United States; Department of Radiation Oncology, University of California Los Angeles, Los Angeles, United States

**Keywords:** NSCLC, clotrimazole, immunotherapy, radiation, veterans

## Abstract

**Background:**

Preclinical data suggest antifungal azole derivatives have antitumor efficacy that may modulate response to immune checkpoint inhibitors (ICIs). We aimed to evaluate the association of azole drugs with overall survival (OS) in a population of patients with non-small cell lung cancer (NSCLC) treated with ICI within the Veterans Health Administration (VHA).

**Methods:**

In this retrospective study, the VA Corporate Data Warehouse was queried for patients diagnosed with NSCLC and treated with ICI from 2010 to 2018. Concomitant oral azole use was defined as dispensation by a VA pharmacy within 90 days of the first ICI infusion. Patients who received azole after 30 days were excluded from the analysis to mitigate immortal time bias. OS was measured from the start of ICI. Cox regression and propensity score matching were used to adjust for confounders.

**Results:**

We identified 3413 patients with NSCLC receiving ICI; 324 (9.5%) were exposed to concomitant azoles. As a group, azole use was not associated with OS (hazard ratio [HR] = 0.96; 95% CI, 0.84-1.09; *P* = .51). After stratification by azole type, clotrimazole had an association with better OS on univariable (HR = 0.75; 95% CI, 0.59-0.96; *P* = .024) and multivariable analysis (HR = 0.71; 95% CI, 0.56-0.91; *P* = .007). Propensity score matching of patients who received clotrimazole vs no azole yielded 101 patients per matched cohort. Clotrimazole was associated with improved OS, although this did not meet the threshold for statistical significance (HR = 0.74; 0.54-1.01; *P* = .058).

**Conclusion:**

This observational study demonstrated an association between clotrimazole and OS among patients with advanced NSCLC receiving ICI. These findings build upon preclinical evidence and support further investigation into the potential for clotrimazole as a repurposed FDA drug to improve cancer outcomes.

## Teaser

This study evaluated the association of azole drugs with overall survival in patients with non-small cell lung cancer treated with immune checkpoint inhibitors.

**Implications for practice**

In this study of patients with non-small cell lung cancer who received immune checkpoint inhibitors, we identified improved overall survival in patients who received concurrent clotrimazole antifungal therapy. Clotrimazole may be an ideal target for drug repurposing to potentially improve the efficacy of immunotherapy and should be further evaluated in a clinical trial.

## Introduction

Lung cancer is the leading cause of cancer mortality in the United States.^1,2^ Immune checkpoint inhibitors (ICIs) have significantly improved overall survival in patients with metastatic and locally advanced non-small cell lung cancer (NSCLC).^3,4^ However, the majority of patients do not benefit and develop disease progression due to a variety of resistance mechanisms.^5^ Preclinical studies have shown promising results with regard to the potential for commonly used drugs to potentiate antitumor immunity in patients when given concomitantly with ICIs.^6^ These include data demonstrating the potential for azole antifungal drugs to inhibit cell proliferation and invasion in vitro.^7,8^ Clotrimazole, specifically, has been found to have antitumor effect in numerous human cancer cell lines, including melanoma, glioma, lung, colorectal, and endometrial cancers.^9-15^ This effect has in part been attributed to its ability to inhibit glycolytic enzymes and calmodulin, causing energy starvation.^16^ Additionally, clotrimazole has been found to have direct effects on improving antitumor immunity through enhancement of dendritic cell antigen presentation and T-cell activation.^17^ Despite these promising preclinical effects, azole drugs have not been studied in human patients receiving immunotherapy. We therefore studied the association of azoles with overall survival in a real-world cohort of patients with NSCLC receiving ICI.

## Methods

We queried the Veterans Health Administration (VHA) Corporate Data Warehouse and identified patients with NSCLC treated with either nivolumab, pembrolizumab, durvalumab, or atezoumab between 2010 and 2018. Receipt of azole drugs was defined as taking an oral azole drug within 90 days of the start of ICI therapy; azole drugs with alternative routes of administration (eg, topical, otic, or intravenous) were excluded. Patients who first received azole after 30 days beyond the start of ICI were excluded from the analysis to mitigate immortal time bias. Pearson’s *χ*^2^ tests were used to assess the associations among patient, disease, and treatment characteristics and azole usage.

Overall survival was measured from the date of the first ICI administration to the date of the last follow-up or death. Kaplan-Meier analysis and univariable Cox regression were performed, with survival expressed as hazard ratios (HR) and 95%CI. Multivariable Cox proportional hazards regression was performed using backward elimination with an alpha level of removal of 0.05. Propensity score matching (1:1, nearest neighbor) was additionally used to adjust for potential confounding. Statistical analyses were performed using SAS Enterprise Guide 7.1 (SAS Institute Inc.). Analyses were performed testing azoles as a group and also by individual azole drugs. Tests were 2-sided with a level of significance of *P* = .05.

## Results

We identified a total of 3413 Veterans with NSCLC treated with ICI, of which 324 (9.5%) received a concomitant azole drug (Table 1). Azole use was associated with a higher proportion of patients with Black race and elevated comorbidity index. Of patients who received azoles, most received clotrimazole (31.2%), followed by fluconazole (26.9%), more than one azole drug (20.1%), ketoconazole (12.7%), miconazole (8.6%), and itraconazole (0.6%).

**Table 1.** Descriptive statistics of the overall cohort.

		Azole				
Variable	Categories	No *N* = 3089		Yes *N* = 324		
		*N*	*%*	*N*	*%*	*P*
Age	≤65	810	26.2	79	24.4	.71
	66-70	973	31.5	100	30.9	
	71-75	733	23.7	86	26.5	
	>75	573	18.6	59	18.2	
Race	White	2233	72.3	254	78.4	.009
	Black	661	21.4	44	13.6	
	Other	42	1.4	20	6.2	
	Unknown	153	5.0	6	1.9	
Gender	Male	3002	97.2	311	96.0	.23
	Female	87	2.8	13	4.0	
Geography	Urban	2022	65.5	225	69.4	.15
	Rural	1067	34.5	99	30.6	
Employment	Employed	622	20.1	69	21.3	.81
	Not employed	1304	42.2	142	43.8	
	Retired	1063	34.4	103	31.8	
	Unknown	100	3.2	10	3.1	
Marital status	Married	1429	46.3	158	48.8	.54
	Not married	1655	53.6	166	51.2	
	Unknown	5	0.2	0	0	
Elixhauser comorbidity index	0-4	906	29.3	65	20.1	<.001
	5-6	692	22.4	63	19.4	
	7-9	815	26.4	81	25.0	
	>9	676	21.9	115	35.5	
Histology	Squamous cell carcinoma	1127	36.5	119	36.7	.97
	Adenocarcinoma	1469	47.6	155	47.8	
	Other	493	16.0	50	15.4	
Stage at diagnosis	0	4	0.1	0	0.0	.11
	I	374	12.1	53	16.4	
	II	222	7.2	29	9.0	
	III	835	27.0	90	27.8	
	IV	1271	41.2	121	37.4	
	Unknown	383	12.4	31	9.6	
Year of diagnosis	2010-2015	1333	43.2	123	38.0	.072
	2016-2018	1756	56.9	201	62.0	
Months from diagnosis to ICI	0-4	737	23.9	85	26.2	.77
	5-10	859	27.8	89	27.4	
	11-19	680	22.0	71	21.9	
	≥ 20	813	26.3	79	24.4	
Chemotherapy	None	408	13.2	44	13.6	.25
	Before ICI	1649	53.4	187	57.7	
	During ICI	956	31.0	89	27.5	
	After ICI	76	2.5	4	1.2	

Abbreviations: ICI, immune checkpoint inhibitor.

**Figure 1.**
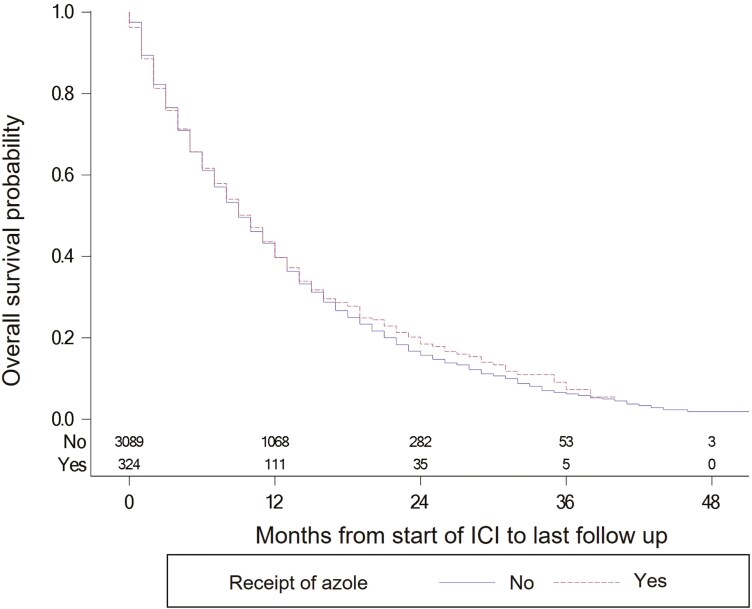
Kaplan-Meier curves for overall survival, comparing patients who received concomitant azole versus those who did not. Hazard ratio = 0.96; 95% CI, 0.84-1.09; *P* = .51.

On univariable analysis, there was no statistically significant difference in overall survival between patients who did and did not receive concomitant azoles (HR = 0.96; 95% CI, 0.84-1.09; *P* = .51) (Figure 1). Median overall survival was 10 and 9 months, respectively; 1-year overall survival was 39.7% (95% CI, 34.0%-45.3%) and 39.8% (95% CI, 37.9%-41.6%), respectively. This was also found in multivariable analysis (HR = 0.92; 95% CI, 0.80-1.05; *P* = .20) (Table S1).

When analyzing individual azoles, clotrimazole was associated with better crude rates of overall survival (HR = 0.75; 95% CI, 0.59-0.96; *P* = .024), as was the receipt of more than one azole (HR = 0.73; 95% CI 0.54-0.97; *P* = .033). Miconazole was associated with worse overall survival (HR = 2.20; 95% CI 1.49-3.24; *P* < .001). On multivariable analysis (Table 2), clotrimazole was the only individual azole to be associated with better overall survival (HR = 0.71; 95% CI, 0.56-0.91; *P* = .007). Receipt of more than one azole remained associated with better overall survival (HR = 0.71; 95% CI, 0.53-0.96; *P* = 0.027) and miconazole remained associated with worse overall survival (HR = 1.91; 95% CI, 1.29-2.82; *P* = .001). Factors associated with better overall survival on multivariable analysis include chemotherapy receipt during or after ICI (vs before), age >70, Black race, female sex, Elixhauser Comorbidity Index 0-4, employed status (vs retired), non-squamous cell histology, non-metastatic cancer, and longer duration from diagnosis to ICI (>19 months vs ≤19 months).

**Table 2.** Multivariable cox regression for overall survival in entire ICI cohort

Variable	Categories	HR	95%CI	*P*
Azole	Itraconazole	3.81	0.94-15.39	.061
	Fluconazole	1.15	0.90-1.47	.25
	Ketoconazole	0.98	0.69-1.39	.89
	Clotrimazole	0.71	0.56-0.91	.007
	Miconazole	1.91	1.29-2.82	.001
	2 + azoles	0.71	0.53-0.96	.027
	No azole	—	—	—
Age	≤65	1.43	1.26-1.63	<.001
	66-70	1.35	1.20-1.52	<.001
	71-75	0.94	0.83-1.07	.35
	>75	—	—	—
Race	White	—	—	—
	Black	0.86	0.77-0.95	.003
	Other	1.00	0.72-1.39	1.00
	Unknown	1.08	0.91-1.29	.37
Gender	Male	—	—	—
	Female	0.75	0.58-0.95	.020
Employment	Employed	—	—	—
	Not employed	1.01	0.91-1.13	.84
	Retired	1.20	1.07-1.34	.002
	Unknown	1.16	0.92-1.47	.22
Elixhauser comorbidity index	0-4	—	—	—
	5-6	1.12	1.01-1.26	.039
	7-9	1.27	1.14-1.41	<.001
	>9	1.21	1.09-1.36	<.001
Histology	Squamous cell carcinoma	—	—	—
	Adenocarcinoma	0.80	0.74-0.88	<.001
	Other	0.91	0.81-1.03	.013
Stage at diagnosis	0	0.48	0.12-1.93	.30
	I	1.00	0.88-1.15	.96
	II	0.94	0.80-1.10	.43
	III	0.81	0.74-0.90	<.001
	IV	—	—	—
	Unknown	1.12	0.087	.087
Months from diagnosis to ICI	0-4	1.43	1.26-1.63	<.001
	5-10	1.28	1.15-1.44	<.001
	11-19	1.17	1.04-1.30	.007
	>19	—	—	—-
Chemotherapy	None	0.95	0.84-1.09	.47
	Before ICI	—	—	—
	During ICI	0.69	0.63-0.75	<.001
	After ICI	0.55	0.42-0.73	<.001

*Note*: Backward selection with an *α* of 0.05 was used. The following variables were removed from the model: year of diagnosis, geography, and marital status.

Abbreviations: HR, hazard ratio; 95%CI, 95% confidence interval; ICI, immune checkpoint inhibitor.

**Figure 2.**
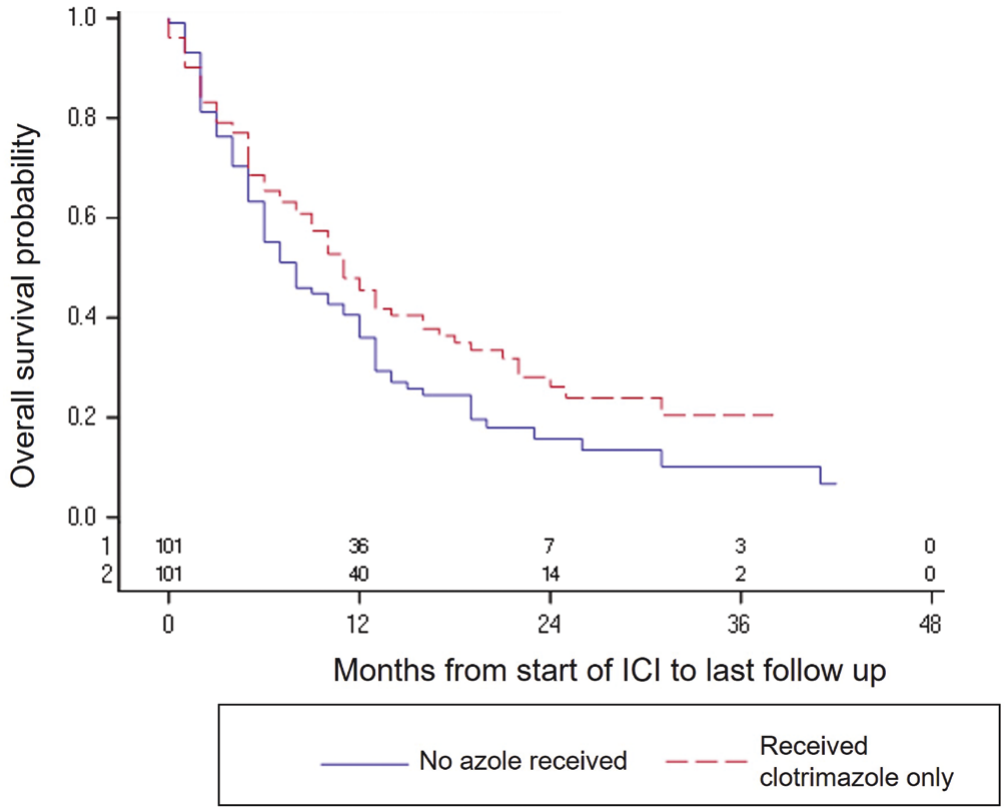
Kaplan-Meier curves for overall survival, comparing propensity score-matched patients who received concomitant clotrimazole vs no azole. Hazard ratio = 0.74; 95% CI, 0.54-1.01; *P* = .058.

Given the unique association of clotrimazole with better overall survival, we performed a multivariable analysis of patients who received clotrimazole vs no azole (Table 3). Receipt of clotrimazole was associated with better OS (HR = 0.71; 95% CI, 0.56-0.91; *P* = .007). Additionally, we performed propensity score matching of patients who received clotrimazole vs no azole, which yielded 101 patients in each cohort well balanced in baseline characteristics (Table S2). Concomitant clotrimazole had a statistically non-significant association with improved overall survival (HR = 0.74; 95% CI, 0.54-1.01; *P* = .058) (Figure 2). Median overall survival was 11 months and 9 months, respectively, for patients who received clotrimazole vs no azole; 1-year overall survival was 45.5% (95% CI, 35.0%-55.4%) and 37.8% (95% CI, 27.6-47.9%), respectively.

**Table 3.** Cox multivariate regression for overall survival in subset analysis of patients who received clotrimazole vs no azole.

Variable	Categories			
		HR	95%CI	*P*
Clotrimazole	No	—	—	—
	Yes	0.71	0.56-0.91	.007
Age	≤65	1.43	1.25–1.63	<.001
	66-70	1.37	1.22–1.55	<.001
	71-75	0.95	0.83–1.08	.40
	>75	—	—	—
Race	White	—	—	—
	Black	0.84	0.76–0.93	<.001
	Other	0.92	0.65–1.31	.66
	Unknown	1.08	0.90–1.30	.39
Gender	Male	—	—	—
	Female	0.74	0.57–0.96	.023
Employment	Employed	—	—	—
	Not employed	1.00	0.90–1.12	1.00
	Retired	1.19	1.06–1.34	.003
	Unknown	1.15	0.90–1.47	.26
Elixhauser comorbidity index	0-4	—	—	—
	5-6	1.11	0.99–1.24	.071
	7-9	1.28	1.15–1.43	<.001
	>9	1.22	1.09–1.37	<.001
Histology	Squamous cell carcinoma	—	—	—
	Adenocarcinoma	0.81	0.74–0.88	<.001
	Other	0.89	0.79–1.01	.066
Stage at diagnosis	0	0.48	0.12–1.95	.31
	I	1.01	0.88–1.17	.84
	II	0.93	0.79–1.10	.42
	III	0.81	0.73–0.90	<.001
	IV	—	—	—
	Unknown	1.12	0.98–1.28	.085
Months from diagnosis to ICI	0-4	1.43	1.26–1.64	<.001
	5-10	1.30	1.16–1.46	<.001
	11-19	1.19	1.06–1.33	.004
	>19	—	—	—
Chemotherapy	None	0.97	0.85–1.11	.65
	Before ICI	—	—	—
	During ICI	0.68	0.62–0.75	<.001
	After ICI	0.55	0.41–0.72	<.001

*Note*: Backward selection with an *α* of 0.05 was used. The following variables were removed from the model: year of diagnosis, geography, marital status.

Abbreviations: HR, hazard ratio; 95%CI, 95% confidence interval; ICI, immune checkpoint inhibitor.

## Discussion

Several studies suggest that azole antifungals may be candidates for drug repurposing given their anticancer effects demonstrated in vitro and in vivo (Table S3). In what is, to the best of our knowledge, the only clinical study evaluating the association of azole antifungals and overall survival in patients receiving ICI, the concomitant receipt of an azole therapy was not associated with better overall survival. However, our analysis demonstrates an association of improved overall survival in patients who received concomitant clotrimazole.

Clotrimazole has been identified as having several mechanisms that exert antitumor effects. In vitro data shows clotrimazole inhibits glycolytic enzymes and glucose uptake in human breast cancer cell lines,^12^ and has shown similar effects in melanoma and lung adenocarcinoma and colon adenocarcinoma.^10,18^ It has also specifically been found to interact with the glycolytic enzyme hexokinase to induce the release of cytochrome c and apoptosis.^19^ It has separately been found to inhibit growth-factor-induced cell proliferation through depletion of intracellular calcium via action on calmodulin, which decreases the metastatic potential of melanoma in vivo.^2^ Clotrimazole’s interaction with hexokinase 2 and regulation of lactate metabolic production has immunomodulatory effects as well, leading to activation of dendritic cell-mediated antigen presentation and potentiation of T-cell response via interaction. In mouse models, clotrimazole increases antitumor immune cell infiltration and enhances the antitumor efficacy of anti-PD1 therapy, and this mechanism in particular may explain our finding of improved overall survival in the setting of ICI.^17^

It is worth noting that itraconazole is another azole derivative with a strong association with antitumor effect via inhibition of the Hedgehog pathway, angiogenesis, endothelial proliferation, cell cycle progression, and chemotherapeutic drug resistance.^20^ Surprisingly, we identified an association of itraconazole with *worse* overall survival, although this was statistically non-significant, perhaps due to the low proportion of patients who received this drug.

Our study identified other covariates associated with survival in Veterans with NSCLC who received immunotherapy. Patients with fewer comorbidities and employed patients had better overall survival, which may reflect better performance status. The association of older age, female sex, Black race, and non-squamous tumor histology with better overall survival may reflect underlying biological differences associated with improved antitumor immunity in the setting of immunotherapy or perhaps decreased risk of immune-mediated adverse effects from immunotherapy.^21-24^ Receipt of chemotherapy during or after ICI (vs before) may reflect treatment with more modern systemic therapy paradigms^25^ or earlier-line therapy. Non-metastatic cancer and a longer duration from diagnosis to ICI indicate less aggressive disease.

There are several limitations of this study inherent to its retrospective design. The VA Corporate Data Warehouse does not contain data on disease progression, which would have been less susceptible to selection bias vs overall survival. It also does not contain information regarding the dosing of specific drugs and cannot account for the lack of consistent use of drugs by patients. Additionally, the proportion of patients taking azoles is quite small, may introduce bias, and limits the statistical power of the analysis (particularly when analyzing individual drugs). We acknowledge that oral clotrimazole is typically administered as a troche lozenge, which may have low systemic concentration. However, it is possible that the serum concentrations required for the immunostimulatory effect are less than for the antifungal effect. Furthermore, it is possible the effect may be mediated in part through interaction with oral/gut microbiome^26^ rather than a direct effect. Clotrimazole’s statistically significant association with overall survival in our study could be due to selection bias. For example, the association may be a surrogate for a more aggressive detection or treatment of fungal infection (eg, oropharyngeal candidiasis) in patients, reflecting a higher quality of care. It is also possible that selection bias results in negative findings in the grouped azole cohort if azole drug therapy is a surrogate for severe fungal infections and immunocompromised, ultimately portending a poor prognosis.

In summary, we studied the azole antifungal drugs in NSCLC patients receiving ICI and found no association with overall survival when analyzing azoles as a group but did identify an association with better overall survival specifically with clotrimazole. These results are hypothesis generating but may indicate clotrimazole has antitumor direct or immune effects that potentiate response to ICI, and further study using an independent cohort is needed.

## Supplementary Material

oyae262_suppl_Supplementary_Tables

## Data Availability

The data underlying this article will be shared on reasonable request to the corresponding author.
