# Supplementary material for: The association of azole antifungals with overall survival in patients with non-small cell lung cancer receiving immune checkpoint inhibitors
Source: Oncologist. 2024 Sep 25;30(2):oyae262. doi: 10.1093/oncolo/oyae262 (PMC11883151; doi:10.1093/oncolo/oyae262)
Supplement: oyae262_suppl_Supplementary_Tables [file oyae262_suppl_supplementary_tables.docx]

**Supplemental Table 1.** Cox Multivariable Regression for Overall Survival in Entire ICI Cohort, with azole drugs categorized as a group

| Variable | Categories |  | UVA | | | | MVA | |  |  | |
| --- | --- | --- | --- | --- | --- | --- | --- | --- | --- | --- | --- |
|  |  | *N* | HR | 95%CI | *p* |  | | HR | 95%CI | *p* |  |
| Azole | no  yes | 3089  324 | --  0.96 | --  0.84 – 1.09 | --  0.51 |  | | --  0.92 | --  0.80 – 1.05 | --  0.20 |  |
| Age | ≤65  66-70  71-75  >75 | 889  1073  819  632 | 1.21  1.24  0.90  -- | 1.08 – 1.36  1.11 – 1.39  0.79 – 1.01  -- | 0.001  <0.001  0.075  -- |  | | 1.44  1.37  0.95  -- | 1.27 – 1.64  1.22 – 1.54  0.84 – 1.07  -- | <0.001  <0.001  0.385  -- |  |
| Race | white  black  other  unknown | 2487  705  48  173 | --  0.86  1.03  1.04 | --  0.78 – 0.95  0.74 – 1.44  0.88 – 1.23 | --  0.002  0.85  0.66 |  | | --  0.85  0.97  1.07 | --  0.77 – 0.94  0.70 – 1.35  0.90 – 1.28 | --  0.002  0.86  0.43 |  |
| Gender | male  female | 3313  100 | --  0.85 | --  0.67 – 1.09 | --  0.20 |  | | --  0.76 | --  0.60 – 0.97 | --  0.030 |  |
| Geography | urban  rural | 2247  1166 | --  1.05 | --  0.97 – 1.14 | --  0.22 |  | |  |  |  |  |
| Employment | employed  not employed  retired  unknown | 691  1446  1166  110 | --  1.06  1.12  1.21 | --  0.95 – 1.17  1.01 – 1.25  0.96 – 1.53 | --  0.30  0.034  0.11 |  | | --  1.01  1.20  1.15 | --  0.91 – 1.13  1.07 – 1.35  0.91 – 1.46 |  |  |
| Marital Status | married  not married  unknown | 1587  1821  5 | --  0.97  1.20 | --  0.90 – 1.04  0.50 – 2.89 | --  0.40  0.68 |  | |  |  |  |  |
| Elixhauser Comorbidity Index | 0-4  5-6  7-9  >9 | 971  755  896  791 | --  1.11  1.32  1.23 | --  1.00 – 1.24  1.19 – 1.46  1.11 – 1.37 | --  0.060  <0.001  <0.001 |  | | --  1.111.27  1.21 | --  1.00 – 1.24  1.15 – 1.42  1.09 – 1.35 | --  0.060  <0.001  <0.001 |  |
| Histology | squamous cell carcinoma  adenocarcinoma  other | 1246  1624  543 | --  0.82  0.96 | --  0.76 – 0.89  0.86 – 1.08 | --  <0.001  0.52 |  | | --  0.80  0.92 | --  0.74 – 0.88  0.82 – 1.03 | --  <0.001  0.14 |  |
| Stage at Diagnosis | 0  I  II  III  IV  unknown | 4  427  251  925  414  1392 | 0.41  0.88  0.87  0.82  --  1.08 | 0.10 – 1.65  0.78 – 1.00  0.74 – 1.01  0.74 – 0.90  --  0.96 – 1.23 | 0.21  0.043  0.070  <0.001  --  0.20 |  | | 0.48  1.00  0.93  0.82  --  1.12 | 0.12 – 1.94  0.87 – 1.14  0.79 – 1.09  0.74 – 0.90  --  0.99 – 1.28 | 0.30  0.96  0.35  <0.001  --  0.073 |  |
| Year of Diagnosis | 2010-2015  2016-2018 | 1456  1957 | 0.82  -- | 0.75 – 0.88  -- | <0.001  -- |  | |  |  |  |  |
| Months from Diagnosis to ICI | 0-4  5-10  11-19  >19 | 822  948  751  892 | 1.42  1.33  1.18  -- | 1.27 – 1.58  1.20 – 1.48  1.05 – 1.31  -- | <0.001  <0.001  0.004  -- |  | | 1.43  1.29  1.17  -- | 1.26 – 1.63  1.15 – 1.44  1.05 – 1.31  -- | <0.001  <0.001  0.005  -- |  |
| Chemotherapy | none  before ICI  during ICI  after ICI | 452  1836  1045  80 | 1.07  --  0.71  0.60 | 0.95 – 1.20  --  0.65 – 0.77  0.46 – 0.78 | 0.26  --  <0.001  <0.001 |  | | 0.97  --  0.69  0.56 | 0.85 – 1.10  --  0.63 – 0.75  0.43 – 0.74 | 0.61  --  <0.001  <0.001 |  |

Abbreviations: HR, hazard ratio; ICI, immune checkpoint inhibitor; MVA, multivariable analysis; UVA, univariable analysis; 95%CI, 95% confidence interval

Note: Backward selection with an α of 0.05 was used. The following variables were removed from the model: year of diagnosis, geography, and marital status

**Supplemental Table 2.** Descriptive Statistics of propensity score matched cohort for the clotrimazole subset analysis, χ^2^

| Variable | Categories | No Azole  *N*= 101 | | Clotrimazole  *N*= 101 | |  |  |
| --- | --- | --- | --- | --- | --- | --- | --- |
|  |  | *N* | % | *N* | % | *p* | *SMD* |
| Age | ≤65  66-70  71-75  >75 | 18  41  26  16 | 17.8  40.6  25.7  15.8 | 20  32  26  23 | 19.8  31.7  25.7  22.8 | 0.48 | 0.05  0.19  0.00  0.18 |
| Race | white  black  other  unknown | 82  11  0  8 | 81.2  10.9  0.0  7.9 | 81  13  2  5 | 80.2  12.9  2.0  5.0 | 0.41 | 0.03  0.06  0.20  0.12 |
| Gender | male  female | 100  1 | 99.0  1.0 | 100  1 | 99.0  1.0 | 1.00 | 0.00  0.00 |
| Geography | urban  rural | 72  29 | 71.3  28.7 | 75  26 | 74.3  25.7 | 0.64 | 0.07  0.07 |
| Employment | employed  not employed  retired  unknown | 25  43  25  8 | 24.8  42.6  24.8  7.9 | 25  40  32  4 | 24.8  39.6  31.7  4.0 | 0.51 | 0.00  0.06  0.15  0.17 |
| Marital Status | married  not married | 55  46 | 54.5  45.5 | 55  46 | 54.5  45.5 | 1.00 | 0.00  0.00 |
| Elixhauser Comorbidity Index | 0-4  5-6  7-9  10+ | 25  18  21  37 | 24.8  17.8  20.8  36.6 | 19  23  24  35 | 18.8  22.8  23.8  34.7 | 0.64 | 0.14  0.12  0.07  0.04 |
| Histology | squamous cell carcinoma  adenocarcinoma  other | 38  48  15 | 37.6  47.5  14.9 | 39  46  16 | 38.6  45.5  15.8 | 0.96 | 0.020  0.040  0.027 |
| Stage at Diagnosis | I  II  III  IV  unknown | 19  7  33  39  3 | 18.8  6.9  32.7  38.6  3.0 | 15  11  27  42  6 | 14.9  10.9  26.7  41.6  5.9 | 0.55 | 0.11  0.14  0.13  0.06  0.14 |
| Year of Diagnosis | 2010-2015  2016-2018 | 40  61 | 39.6  60.4 | 38  63 | 37.6  62.4 | 0.77 | 0.04  0.04 |
| Months from Diagnosis to first Chemo* | 0-4  5-10  11-19  ≥ 20 | 18  28  31  24 | 17.8  27.7  30.7  23.8 | 21  32  26  22 | 20.8  31.7  25.7  21.8 | 0.80 | 0.08  0.09  0.11  0.05 |

**Supplemental Table 3.** Select preclinical studies evaluating antitumor effect of azole antifungals

|  | **Reference** | **Proposed Drug Mechanism of Action** |
| --- | --- | --- |
| **Clotrimazole** | Wang, J Immunother Cancer, 2021 | Triggers dendritic cell activation via lactate-lysosome axis and promotes antigen presentation |
|  | Furtado, PLoS One, 2012 | Reduces glucose uptake in a dose-dependent manner and inhibited glycolytic enzymes in breast cancer cell lines |
|  | Penso, Eur J Pharmacol, 2002. | Reduces glycolysis and levels of ATP in lung carcinoma and colon adenocarcinoma cell lines via inhibition of calmodulin |
|  | Penso, Eur J Pharmacol, 1998 | Induces dose-dependent detachment of glycolytic enzyme hexokinase from the mitochondria of B16 melanoma cells, reducing cell viability |
|  | Chiara, PLoS One, 2008 | Induces detachment of hexokinase II from mitochondria and led to concentration-dependent cell death in HeLa cells |
|  | Benzaquen, Nature, 1995 | Depletes intracellular calcium and inhibits cell proliferation of several cancer cell lines and inhibits in vivo formation of melanoma lung metastases |
|  | Khalid, J Neurosurg, 2005 | Inhibits cell growth of glioma cells lines, which could not be overcome with exogenous epidermal growth factor, and prevented brain tumor formation in rate brains |
|  | Song, Sci Rep, 2024 | Promotes ROS-mediated apoptosis, G0/G1 arrest, and NF-ΚΒ pathway inhibition in multiple myeloma |
| **Itraconazole** | Wang, Biomed Pharmacother, 2020 | Inhibits cell growth and induces apoptosis in hepatocellcular carcinoma cell lines via action on Wnt, PI3K, and ROS pathways |
|  | Chen, Mol Cancer Ther, 2018 | Activates AMPK signaling and inhibits survival and proliferation of human esophageal cancer cell lines |
|  | Zhang, Mol Cancer Ther, 2021 | suppresses HER2/AKT pathway signaling in esophageal cancer cell lines and xenografts and showed reduction in tumor HER2 expression in a pilot phase I clinical trial in humans |
|  | Chong, ACS Chem Biol, 2007 | Inhibits endothelial cell cycle progression and blocks vascular endothelial growth factor-dependent angiogenesis in vivo |
|  | Kim, Cancer Cell, 2010 | Suppresses the Hedgehog signaling pathway and suppresses growth of melanoma in murine xenograft model |
|  | Aftab, Cancer Res, 2011 | Reduces HIF-1 mediated angiogenesis and in vivo tumor vascularity of non small cell lung cancer |
|  | Wang, Cancer Lett, 2017 | Cytotoxic to breast cancer cell lines via reduction of BCL-2 |
|  | Jiang, J Cell Biochem, 2019 | Inhibits proliferation of pancreatic cancer cells via Bak-1 activation in vitro and in xenografts |
| **Miconazole** | Scatozza, Int J Mol Sci, 2024 | Reduces melanoma cell line proliferation via effect on mitochondria-dependent apoptosis |
|  | Wu, Toxicol Appl Pharmacol, 2002 | Induces G0/G1 arrest and apoptosis in colon adenocarcinoma in vitro and in vivo |
|  | Yuan, Oncol Rep, 2017 | Activates mitochdonrial- and death receptor-mediated apoptosis in breast cancer cells |
|  | Yoon, Cancer Sci, 2020 | Suppresses STAT3 activation in lung cancer vitro and in vivo |
| **Ketoconazole** | Forgue-Lafitte, Cancer Res, 1992 | Induces G0/G1 phase arrest in colon and breast cancer cell lines via action on cytochrome P-450 |
|  | Chen, Toxicol Appl Pharmacol, 2000 | Induces G0/G1 arrest in colorectal and hepatocellular cancer cell lines |
|  | Chen, J Hepatol, 2019 | Enhances PINK1/Parkin-mediated mitophagy in hepatocellular carcinoma by downregulation of COX-2 |
|  | Agnihotri, Clin Cancer Res, 2019 | Inhibits glioblastoma cells in vitro and in vivo via alteration of hexokinase 2-regulated pathways |
